# How Can Psychiatrists Care for the Brain, Mind and Soul of their Patients?

**DOI:** 10.1192/j.eurpsy.2023.2071

**Published:** 2023-07-19

**Authors:** M. Sidhu

**Affiliations:** Psychiatry, Cambridgeshire & Peterborough NHS Foundation Trust, Peterborough, United Kingdom

## Abstract

**Introduction:**

Neuroscientific theories define the brain as a physical organ that provides the electrochemical basis for cognitive, sensory and motor functions; psychological theories define the mind as the hub of consciousness; and spiritual theories define the soul as our indivisible essence of being. We lack clarity on the mechanisms by which physiological processes in the brain give rise to mental phenomena in the mind, while the concept of the soul lacks scientific grounding. However, frameworks for understanding the brain, mind and soul have an impact on the aetiology, diagnosis, and treatment of mental illnesses, thereby shaping clinical practice.

**Objectives:**

To review the current literature explaining how psychiatrists can care for the brain, mind and soul of their patients.

**Methods:**

Six electronic databases were searched for articles explaining how psychiatrists can care for the brain, mind and soul of their patients. Other relevant papers identified from the reference lists of included articles were also selected. A narrative literature review was undertaken due to the heterogeneity of the articles.

**Results:**

Twenty-six articles were included. These revealed that a psychiatrist’s approach to care is shaped by their understanding of the roles that the brain, mind and soul play in mental illness. A growing understanding of the brain’s physiological changes in mental illness has enabled the development of targeted electroconvulsive and pharmacological therapies for treatment and quantitative imaging for monitoring. Similarly, identifying and understanding cognitive-behavioural patterns associated with mental illness enables psychopathology to be directly addressed with focussed psychotherapy. Furthermore, helping patients to develop a spiritual connection with themselves, others or a higher power improves several protective factors, such as hopefulness, positive self-conceptions and social participation.

The weight a psychiatrist places on the physiological, psychological and spiritual components of mental illness inevitably influences their choice of treatments. It is important for psychiatrists to reflect on their own beliefs, and to empathise with the views of patients without imposing their own. Open dialogue with patients about their perspectives on each dimension facilitates engagement, empowerment and treatment adherence.

**Conclusions:**

Distinguishing between pathophysiology of the brain, traumatic experiences of the mind, and unmet spiritual needs of the soul enables psychiatrists to accurately identify pathology and personalise care effectively. Further research into the relationships between each component would open additional avenues for understanding and caring for mental illness. As psychiatry strives to address its perceived overreliance on biological therapies, an appreciation for the distinct qualities of the brain, mind and soul has the potential to foster a more patient-centred and holistic approach to care.

**Disclosure of Interest:**

None Declared

